# Transcriptomic and Metabolomic Analysis Reveals Molecular Mechanism of Oxygen-Rich Vacancy Bi_2_MoO_6_ Photocatalytic Inactivation of MRSA

**DOI:** 10.3390/biology15130993

**Published:** 2026-06-24

**Authors:** Runze Zhang, Zhendong Xu, Lin Han, Shuai Qiu, Daxun Li, Hui Bai, Xin Meng, Hua Li, Yunfeng Qi

**Affiliations:** College of Life Sciences, Jilin Normal University, Siping 136000, China; zrzls0109@163.com (R.Z.); 13297386115@163.com (Z.X.); hanlin000516@163.com (L.H.); 18843735062@163.com (S.Q.); 15043217365@163.com (D.L.); 15590693135@163.com (H.B.); 18624395651@163.com (X.M.)

**Keywords:** methicillin-resistant *Staphylococcus aureus*, Bi_2_MoO_6_, transcriptomics, metabolomics, photocatalytic inactivation

## Abstract

Antibiotic-resistant bacteria are widely distributed and threaten public health. Photocatalytic antimicrobial technology can effectively inactivate multidrug-resistant bacteria without readily inducing resistance. We previously showed that oxygen-rich vacancy Bi_2_MoO_6_ (OBM) exhibits excellent activity against methicillin-resistant *Staphylococcus aureus* (MRSA), but the underlying molecular mechanisms remain poorly understood. Here, we employed integrated transcriptomics and metabolomics, with qRT-PCR validation, to systematically elucidate the antibacterial mechanism of OBM against MRSA. OBM treatment induced profound transcriptional and metabolic alterations: 231 differentially expressed genes and 206 differentially abundant metabolites were identified. Functional enrichment analysis revealed cooperative involvement in multiple critical pathways, including inhibition of amino acid biosynthesis and protein translation, disruption of cell wall and membrane integrity, induction of oxidative stress, collapse of energy metabolism (suppression of oxidative phosphorylation and impaired ATP synthesis), and imbalance in nucleotide metabolism (down-regulation of DNA helicase and mismatch repair genes, dysregulation of purine/pyrimidine metabolism). These findings demonstrate that OBM photocatalytically inactivates MRSA through a multi-target systemic attack at both the transcriptional and metabolic levels, providing a novel theoretical foundation for the development of photocatalytic materials aimed at controlling MRSA and other drug-resistant bacteria.

## Simple Summary

Infections caused by methicillin-resistant *Staphylococcus aureus* are difficult to treat and pose a serious threat to public health. Photocatalytic materials can kill such drug-resistant bacteria without easily inducing resistance, but the ways they work at the molecular level are still unclear. In this study, we examined how a bismuth molybdate material with oxygen-rich vacancies destroys methicillin-resistant *Staphylococcus aureus*. By simultaneously analyzing all gene activities and small molecules inside the bacterial cells, we found that the material caused widespread disruption. A total of 231 genes and 206 metabolites changed significantly. These changes blocked the production of amino acids and proteins, damaged the cell wall and membrane, caused oxidative stress, shut down energy supply, and created an imbalance in the building blocks of DNA, leading to errors in DNA copying and repair. Together, these attacks caused a complete and irreversible collapse of the bacterium. Our findings reveal a multi-target killing mechanism that makes resistance unlikely. This new understanding can help scientists design safer and more effective photocatalytic materials to control drug-resistant infections, thereby benefiting public health.

## 1. Introduction

In recent years, methicillin-resistant *Staphylococcus aureus* (MRSA) remains a major global health threat and remains a high priority category in the 2024 World Health Organization list of bacterial priority pathogens, as one of the leading causes of infections in healthcare institutions and communities worldwide [1,2,3]. In our previous study, bismuth molyblate was modified to enhance its performance in photocatalytic inactivation of MRSA. Antibacterial mechanism analysis showed that MRSA was effectively inactivated through the oxidative process of ROS (•O^2−^, •OH, ^1^O_2_), which led to damage to the cell wall and cell membrane [4,5,6]. At present, there are few reports on the photocatalytic inactivation of MRSA by bismuth molybdate, and the mechanism of action on MRSA is not clear, which limits its application in the biomedical field. However, the underlying mechanism of oxygen-enriched bismuth molybdoylate (0.2 Ov-Bi_2_MoO_6_) photocatalytic inactivation of MRSA cannot be fully elucidated at the cellular level. In order to promote the application of bismuth molybdate in the biomedical field, molecular biological methods can be used to further reveal the photocatalytic inactivation mechanism of bismuth molybdate on MRSA.

Transcriptomics is one of the important means to study the phenotype and function of cells. When an organism is stimulated by the outside world, it will immediately transmit signals to the cells, and then activate various stress responses in the body. This process is first carried out through the regulatory instructions of genes [7,8]. RNA is an information carrier for genome transcription and translation, which can accurately control the expression of proteins and then exercise related biological functions. Metabolomics is another new research method after proteomics, which is to study the overall overview of metabolites existing in biological systems under specific conditions and time. At present, metabolomics has been widely used in the study of the response of microorganisms to various environmental stresses or heavy metals, temperature and antibacterial agents [9,10,11,12]. For example, Xu et al. used transcriptomics to explain the molecular mechanism of *E. coli* inactivation by TiO_2_-Ag-AgCl composite antibacterial material, Wu et al. used transcriptomics and metabolomics to analyze the molecular mechanism of E. coli and K. rhizophila inactivated by pH-dependent graphene quantum dots. Du et al. studied the molecular biological mechanism of the visible light-driven carbon/oxygen-doped g-C_3_N_4_ activating persulfate to effectively inactivate antibiotically resistant bacteria based on transcriptomics [13,14,15]. It can be seen that transcriptomics and metabolomics are gradually playing an important role in the study of antibacterial mechanisms, which can provide more reliable and comprehensive information for comprehensively analyzing the molecular mechanism of antibacterial action.

Among various semiconductor materials, perovskite-like structured semiconductors represented by bismuth molybdate (Bi_2_MoO_6_) have emerged as promising candidates for biomedical and environmental applications. Compared to traditional wide-bandgap semiconductors (such as TiO_2_ and ZnO) that primarily respond to UV light, Bi_2_MoO_6_ possesses a narrower bandgap, conferring it with superior visible-light responsiveness and robust structural stability [16]. However, pristine Bi_2_MoO_6_ is often hindered by the rapid recombination of photogenerated electron–hole pairs, which limits the continuous generation of reactive oxygen species (ROS). Defect engineering, particularly the introduction of oxygen vacancies, has been proven to be a highly effective strategy to alter the band structure. These vacancies serve as electron-trapping centers to significantly prolong the lifetime of charge carriers and enhance photocatalytic performance [17,18].

In recent years, transcriptomics and metabolomics data analysis have begun to be applied to assess the biocompatibility and antibacterial deep mechanisms of perovskites and their derived materials. For instance, the multi-omics approach to dissecting the material’s remodeling effect on the metabolic flux of organisms has become a new standard for evaluating the performance of new photocatalysts. However, most current studies on the antibacterial properties of perovskite-like materials still remain at the surface level of ROS generation and physical damage to the cell membrane, lacking systematic analysis of the response mechanism of drug-resistant bacteria (such as MRSA) at the whole-genome and whole-metabolome levels [19,20]. This limits our understanding of the essential differences between photocatalytic inactivation and traditional biochemical inhibition at the molecular level. Therefore, the aim of this study is to investigate the transcriptional regulation and metabolite levels of intracellular mRNA in MRSA treated with 0.2 Ov-Bi_2_MoO_6_ by transcriptome and metabolomics analysis, explore the key metabolic pathways and potential targets, and further clarify the molecular mechanism of 0.2 Ov-Bi_2_MoO_6_ on MRSA, ultimately providing a novel alternative strategy for the treatment of MRSA infections.

## 2. Materials and Methods

### 2.1. Reagents

Tryptone soy broth (TSB) and PBS buffer (pH = 7.3) were purchased from Haibo Biotechnology Co., Ltd., Qingdao, China. The column bacterial total RNA extraction and purification kit and SGExcel FastSYBR Mixture were purchased from Sangon Bioengineering Co., Ltd., Shanghai, China. PrimeScript™ RT reagent Kit with gDNA Eraser (Perfect Real Time) was purchased from Baori Doctor Material Technology Co., Ltd., Beijing, China. Total glutathione peroxidase detection kit, total SOD activity detection kit and lipid oxidation (MDA) detection kit were purchased from Shanghai Biyuntian Biotechnology Co., Ltd., Shanghai, China. 2-nitrophenyl-β-D-galactoside pyranoside (ONPG) was purchased from Shanghai Aladdin Biochemical Technology Co., Ltd., Shanghai, China. MRSA was obtained from the American Center for Bacterial Preservation and deposited in the College of Life Sciences, Jilin Normal University, China. All materials were of analytical-grade purity and could be used without further purification. Distilled water was used in all experiments.

### 2.2. Catalyst Preparation and Biological Sample Collection

Bulk Bi_2_MoO_6_ was first synthesized via a solvothermal route. Briefly, 4 mmol Bi(NO_3_)_3_·5H_2_O was dissolved in 40 mL ethylene glycol, while 2 mmol Na_2_MoO_4_·2H_2_O was separately dissolved in 10 mL ethanol under continuous stirring. The two resulting solutions were mixed and transferred into a 100 mL Teflon-lined autoclave, followed by solvothermal treatment at 190 °C for 2 h. After naturally cooling to ambient temperature, the precipitates were collected by centrifugation, washed three times with ethanol and deionized water sequentially, and vacuum-dried at 70 °C for 12 h to obtain pristine Bi_2_MoO_6_ powders. To fabricate oxygen-vacancy-rich photocatalysts, a classical alkali etching strategy was employed. The as-prepared Bi_2_MoO_6_ was dispersed into NaOH aqueous solutions with concentrations of 0.1, 0.2, 0.3, and 0.4 mol L^−1^. The suspensions were magnetically stirred at room temperature for 20 h. Afterwards, the products were rinsed four times with deionized water and vacuum-dried at 40 °C for 20 h. The corresponding samples were denoted as 0.1 Ov-Bi_2_MoO_6_, 0.2 Ov-Bi_2_MoO_6_, 0.3 Ov-Bi_2_MoO_6_, and 0.4 Ov-Bi_2_MoO_6_. Based on our preliminary antibacterial screenings, 0.2 Ov-Bi_2_MoO_6_ exhibited the optimal photocatalytic bactericidal activity and was thus selected as the primary material for the subsequent evaluations.

All glassware used in the photocatalytic antibacterial experiments was autoclaved prior to use. MRSA was inoculated in tryptone soy broth (TSB, liquid medium) and cultured to the logarithmic phase (approximately 10^7^ CFU mL^−1^) at 37 °C for subsequent experiments. The microbial suspension (200 µL) was added to a quartz bottle containing PBS solution (10 mL), and the 0.2 Ov-Bi_2_MoO_6_ powder was added to each quartz bottle as the experimental group (Group B). The quartz bottles were placed in a PCX-50C multi-channel photochemical reaction system (Bofeilai Technology Co., Ltd., Beijing, China). The photocatalytic inactivation was driven by a 10 W LED lamp with a wavelength range of 400–800 nm (visible light), and the light intensity was strictly calibrated to 100 mW cm^−2^. Importantly, to completely exclude the potential interference of photothermal effects on bacterial viability, the ambient temperature of the reaction system was strictly maintained at 30 °C throughout the irradiation process. The distance between the LED lamp and the quartz bottle was fixed at 10 cm to maintain a homogeneous light intensity of 100 mW cm^−2^ (measured by an optical power meter). The reaction suspension was continuously stirred at 300 rpm using a magnetic stirrer to ensure uniform catalyst distribution. All quartz bottles (capacity 15 mL) were autoclaved before use. After 3 h of treatment, the cells were collected by centrifugation at 8000 r/min for 10 min at 4 °C, washed 3 times with precooled sterile PBS, and frozen in liquid nitrogen. The control group (Group A) was treated with the exact same method (without 0.2 Ov-Bi_2_MoO_6_, but under identical light and thermal conditions). Experiments were repeated six times independently with six biological replicates per group for transcriptomic and metabolomic analyses.

### 2.3. Determination of Oxidative Stress

The activity of total glutathione peroxidase, total superoxide dismutase (SOD) and lipid oxidation (MDA) antioxidant enzymes were measured by microplate reader. β-galactosidase activity was measured by ONPG method. (Additional determination steps as appropriate).

### 2.4. Total RNA Isolation, Library Construction and Transcriptome Data Analysis

#### 2.4.1. Total RNA Extraction

Total bacterial RNA was extracted according to the instructions of the column bacterial total RNA extraction and purification kit. The specific steps were as follows: Bacterial pellets were collected from 5 mL of culture (OD_600_ ≈ 1.0, corresponding to ≈ 10^9^ CFU mL^−1^). After centrifugation (8000 rpm, 10 min, 4 °C), the pellets were resuspended in 100 μL of TE buffer containing 3 mg mL^−1^ lysozyme and incubated at 37 °C for 10 min. Then total RNA was extracted as follows: 100 µL suspension precipitation of 3 mg/mL lysozyme was taken, and the sample was enzymatically hydrolyzed for 10 min at room temperature. Next, 350 µL Buffer Rlysis-BG was added, shaken and mixed immediately, and left for 3 min at room temperature. Following this, 1/2 volume of absolute ethanol was added to the lysed sample and thoroughly mixed. The adsorption column was put into the collection tube, and all the solution was added to the adsorption column with a pipettor, left for 1 min, centrifuged at 12,000 rpm at room temperature for 1 min, and the waste liquid in the collection tube was poured out. The adsorption column was put back into the collection tube, 500 µL GT Solution was added, left for 1 min, centrifuged at 10,000 rpm at 4 °C for 1 min, and the waste liquid in the collection tube was poured out. The adsorption column was put back into the collection tube, 500 µL NT Solution was added, left for 2 min, centrifuged at 10,000 rpm at 4 °C for 1 min, and the waste liquid in the collection tube was poured out. The adsorption column was placed back into the collection tube and centrifuged at 12,000 rpm for 2 min at 4 °C. The adsorption column was placed into an RNase-free 1.5 mL centrifuge tube, and 30 to 50 µL DEPC-treated ddH2O was added to the center of the adsorption membrane, left for 2 min, and centrifuged at 12,000 rpm for 2 min at 4 °C. The resulting RNA solution was stored at −80 °C or used for subsequent tests.

#### 2.4.2. Library Construction and Sequencing

For prokaryotic samples, the library building starting RNA was total RNA, and mRNA was passed through the probe to remove rRNA from total RNA. The resulting mRNA was subsequently randomly interrupted with divalent cations in Fragmentation Buffer. Using fragmented mRNA as template and random oligonucleotides as primers, the first strand of cDNA was synthesized in the M-MuLV reverse transcriptase system, followed by RNA strand degradation by RNaseH and DNA polymerase I system. The second strand of cDNA was synthesized from dNTPs, where dUTP was used instead of dTTP. The purified double-stranded cDNA was end repaired, an A-tail was added and connected to A sequencing adapter. After fragment screening, USER enzyme digestion, amplification and purification, the library was finally obtained.

After the library inspection was qualified, different libraries were pooled according to the requirements of effective concentration and target amount of data, and then sequenced.

#### 2.4.3. Transcriptome Data Analysis

**Figure 1:**
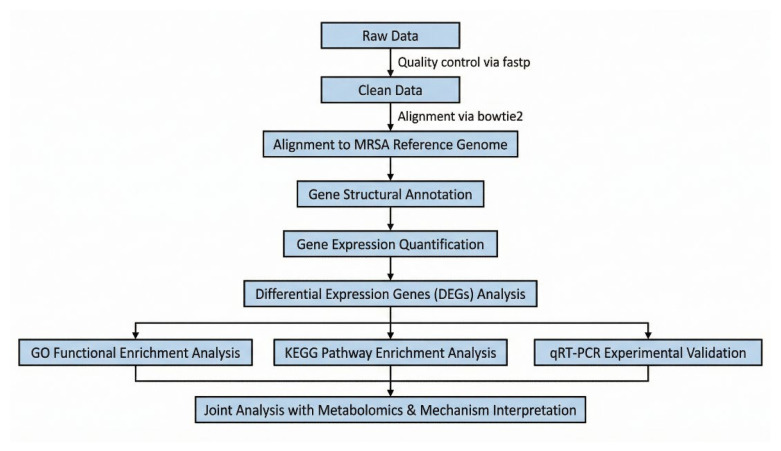
Flowchart of transcriptome data analysis.

The workflow of transcriptome data analysis is shown in Figure 1. Fastp software v0.26.0 was used for quality control processing of raw data for sequencing data of samples to obtain clean data for downstream analysis. The clean data reads after quality control were aligned to the reference genome (*Staphylococcus aureus* subsp. Aureus MRSA252 methicillin-resistant) using bowtie2 software v2.5.4. Next, structural annotation, gene expression analysis, and gene function annotation of related genes were performed.

### 2.5. qRT-PCR Verification

Nine DEGs were randomly selected for qRT-PCR using 16S rRNA as the reference gene to verify the results of RNA-Seq. Primers were designed using Primer Premier 5.0 software. Primer sequences are shown in Table S1.

Total RNA was extracted according to the instructions of the column bacterial total RNA extraction and purification kit. A total of 1 μL PrimeScript RT Enzyme Mix I, 1 μL RT Primer Mix (4×), 4 μL 5× PrimeScript Buffer 2 for Real Time, and 4 μL RNase Free were used, dH_2_O was added to the prepared RNA samples, and the reaction was performed at 35 °C for 15 min and 85 °C for 5 s to complete reverse transcription.

qRT-PCR was performed using an Applied Biosystems 7300/7500 Real Time PCR System (Applied Biosystems, Foster City, CA, USA). A total of 10 μL TB Green Premix Ex Taq II (Tli RNaseH Plus) (2×), 0.8 μL PCR Forward Primer (10 μM), and 0.8 μL PCR Reverse Primer (10 μM), 0.4 μL ROX Reference Dye or Dye II (50×), 2 μL RT reaction solution (cDNA solution) and 6 μL sterilized water were used to form a 20 μL reaction system. PCR amplification program: predeformation at 95 °C for 30 s; 40 cycles were performed at 95 °C for 5 s and 60 °C for 31 s. Each sample was repeated three times.

### 2.6. Metabolite Extraction, LC-MS Analysis and Metabolomic Data Analysis

#### 2.6.1. Metabolite Extraction

For each sample (bacterial pellet from 5 mL culture), 300 μL of ice-cold 80% (*v*/*v*) methanol–water was added. The mixture was immediately flash-frozen in liquid nitrogen for 5 min, then thawed on ice. This freeze–thaw cycle was repeated twice. After the final thaw, the mixture was vortexed for 30 s and sonicated in an ice-water bath for 6 min (ultrasonic power 40 kHz). The lysate was centrifuged at 5000 rpm for 1 min at 4 °C, and the supernatant was lyophilized. The dried residue was reconstituted in 100 μL of 10% (*v*/*v*) methanol–water and filtered (0.22 μm) before LC-MS injection.

#### 2.6.2. LC-MS Analysis

Chromatographic conditions: Chromatography column: Hypersil Gold column (C18); column temperature: 40 °C; flow rate: 0.2 mL/min; mobile phase A: 0.1% formic acid; and mobile phase B: Methanol.

Ms conditions: the scanning range was *m*/*z* 100–1500. ESI source settings are as follows: Spray voltage: 3.5 kV; sheath gas flow rate: 35 psi; aux gas flow rate: 10 L/min; capillary temp: 320 °C; S-lens RF level: 60; aux gas heater temp: 350 °C; polarity: positive, negative; and MS/MS secondary scans were data-dependent scans.

#### 2.6.3. Analysis of Metabolomic Data

**Figure 2:**
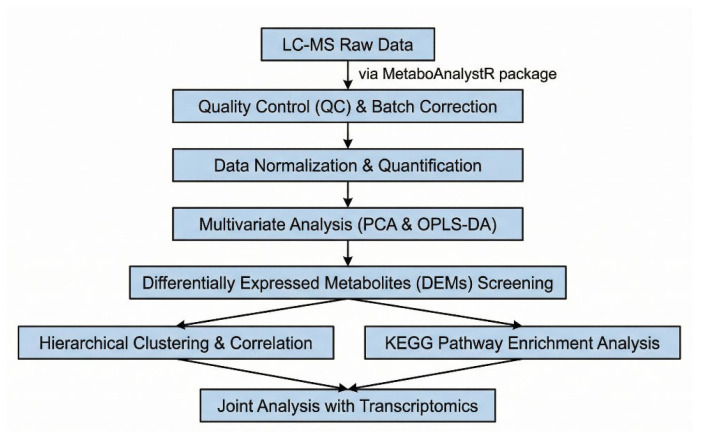
Flowchart of metabolome data analysis.

The metabolome analysis process was based on the R language MetaboAnalystR package v4.0.0. Figure 2 shows the data analysis process of metabolomics, which mainly includes QC quality control of sample data and batch correction, standardization of sample data, metabolite content statistics, unsupervised dimension reduction analysis (PCA), screening of characteristic metabolites (biomarker), correlation analysis and pathway analysis of characteristic metabolites.

#### 2.6.4. Functional Enrichment Analysis

To further explore the changes in the main biological processes and metabolic pathways of *Staphylococcus aureus* cells treated with XJS01, KEGG enrichment analysis was performed for all DEGs and differentially accumulated metabolites (DAMs) using KOBAS 2.0 with default settings. In addition, FDR values were calculated to correct for p-values obtained from independent statistical hypothesis testing. When FDR < 0.05, the pathway term was identified as the target.

#### 2.6.5. Schematic Diagram Generation

All schematic diagrams illustrating the molecular antibacterial mechanisms were generated using Gemini 3.1 Flash Image. Briefly, hand-drawn rough sketch drafts containing core biological pathways, gene regulatory relationships and intracellular structural layouts were manually drawn in advance as structural guidance. A separately prepared reference image with consistent illustration style was uploaded to define uniform line thickness, color palette, layout logic and scientific drawing aesthetic standards. The two types of images were simultaneously imported into the GenAI tool together with descriptive text prompts specifying MRSA metabolic pathways, photocatalytic stress response and multi-target bactericidal cascades. Iterative generation and minor manual post-adjustment were conducted to guarantee scientific accuracy of all pathway and structural information presented in the final figures.

## 3. Results

### 3.1. Characterization of Oxygen Vacancies and Band Structure of 0.2 Ov-Bi_2_MoO_6_

The 0.2 Ov-Bi_2_MoO_6_ photocatalyst used in this study was prepared and characterized in our previous work [1]. Here we summarize the key features relevant to the photocatalytic mechanism.

Oxygen vacancies. Electron paramagnetic resonance (EPR) showed a strong symmetric signal at g ≈ 2.003 (Figure S5c), characteristic of oxygen vacancies. The signal intensity of 0.2 Ov-Bi_2_MoO_6_ was markedly higher than that of pristine Bi_2_MoO_6_, confirming abundant vacancy generation. High-resolution O 1s XPS (Figure S5f) was deconvoluted into three peaks: lattice oxygen (O_1_, 530.03 eV), oxygen vacancies (Ov, 530.63 eV), and adsorbed H_2_O (531.4 eV). The relative area ratio of the Ov peak to the total O 1s signal was calculated to be approximately 18% (a quantitative measure of oxygen vacancy concentration).

Band structure. The UV-vis diffuse reflectance spectrum (Figure S6a) revealed that 0.2 Ov-Bi_2_MoO_6_ has an absorption edge at ≈470 nm, corresponding to a bandgap (Eg) of 2.61 eV (Figure S6b). Mott–Schottky measurements (Figure S7) gave a flat-band potential of −0.57 V vs. NHE, which is taken as the conduction band (CB) edge for this n-type semiconductor. Accordingly, the valence band (VB) edge is located at +2.04 V vs. NHE (CB + Eg). These positions are thermodynamically sufficient to generate •O_2_^−^ (from O_2_/O_2_^−^, −0.33 V) and •OH (from OH^−^/•OH, +1.99 V), consistent with the radical trapping and EPR results presented below (Section 3.2).

### 3.2. Biochemical Characteristics (MDA, GSH-Px, SOD, β-Galactosidase)

**Figure 3:**
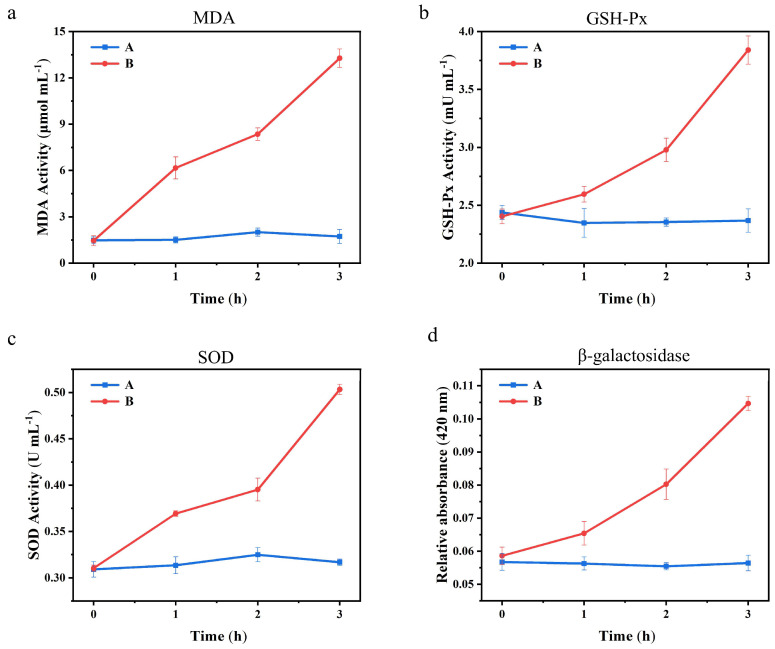
Time-dependent changes of intracellular biochemical indexes in MRSA after 0.2 Ov-Bi_2_MoO_6_ photocatalytic treatment under visible light. (**a**) Malondialdehyde (MDA) content reflecting lipid peroxidation level; (**b**) total glutathione peroxidase (GSH-Px) activity; (**c**) total superoxide dismutase (SOD) activity; and (**d**) intracellular leakage of β-galactosidase representing cell membrane damage. Group A: blank control (light only without catalyst); Group B: experimental group treated with 0.2 Ov-Bi_2_MoO_6_ + visible light.

The ROS produced by the photocatalytic system may damage the cell membrane and cause a stress response, thereby accelerating bacterial inactivation and intracellular DNA leakage. As shown in Figure 3a, MDA content in the experimental group (B) increased linearly with the extension of treatment time, while MDA content in the control group (A) remained unchanged, indicating that the photocatalytic system in the experimental group (B) continuously produced ROS, attacked the polyunsaturated fatty acids of the cell membrane, and lipid peroxidation was significantly amplified, indicating that the cell was in a typical oxidative stress state. As shown in Figure 3b,c, the activities of GSH-Px and SOD also increased linearly in experimental group (B), which may mean that bacteria want to eliminate the toxic effects of ROS, thereby reducing oxidative stress and protecting cells from damage. This evidence suggests that bacterial inactivation is likely due to ROS-induced oxidative inactivation of the cells. The integrity of the bacterial cell wall and the permeability of the cell membrane were evaluated by β-galactosidase activity. As shown in Figure 3d, the β-galactosidase content in the experimental group (B) increased steadily, indicating that the bacterial cell wall and cell membrane may be damaged, leading to a large amount of β-galactosidase leakage, thereby inhibiting the formation of a MRSA biofilm and leading to the death of MRSA.

### 3.3. Transcriptome Analysis

#### 3.3.1. Analysis of Differentially Expressed Genes

**Figure 4:**
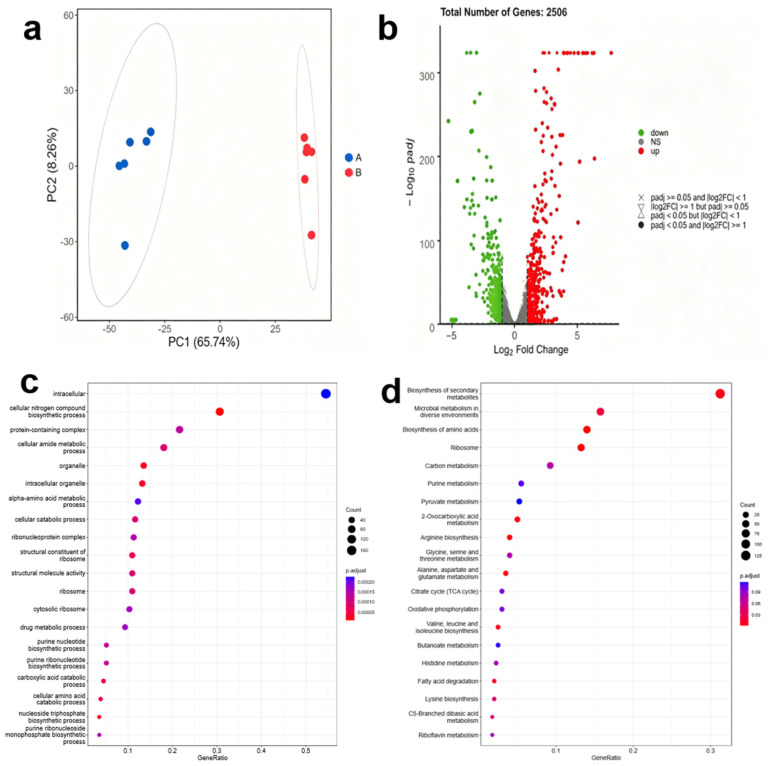
Comprehensive transcriptomic analysis of MRSA with/without 0.2 Ov-Bi_2_MoO_6_ treatment. (**a**) PCA score plot showing distinct separation between control group (A) and treated group (B); (**b**) volcano plot displaying up-regulated (red) and down-regulated (blue) differentially expressed genes (DEGs); (**c**) top enriched GO functional classification of all DEGs; and (**d**) bubble diagram of significantly enriched KEGG metabolic pathways for DEGs.

We obtained high-quality transcriptome data from 12 samples, with a total of 26.66 G of clean data, more than 1.85G of Q30 bases per sample, more than 97.28%, and GC content ranging from 34.26% to 36.631% (Table S2). The majority of clean readings, ranging from 96.07% to 97.65%, were successfully mapped to the MRSA genome (Table S3). The results of the principal component analysis (PCA) showed that the two principal components (PC1, PC2) could effectively distinguish the control group (Group A) and the experimental group (Group B), and the samples within the group were clustered, while the samples between the groups were scattered and very different (Figure 4a). In addition, a heat map of expression correlations between samples showed strong within-group correlations in the selected samples, exceeding a threshold of 0.9 (Figure S1a). Expressed gene count identified 2627 genes expressed in MRSA, of which 889 (33.84%) were DEGs compared to controls (Figure 4b), of which 503 were up-regulated and 386 were down-regulated (Figure S1b).

#### 3.3.2. Enrichment Analysis

To gain a detailed understanding of DEG function, GO and KEGG enrichment analyses were performed (Figure 4c,d). There were 385 GO terms in 2627 DEGs, including 245 (63.64%) biological process terms, 30 (7.79%) cell component terms and 110 (28.57%) molecular function terms. In GO terms, its biological processes include “cellular nitrogen compound biosynthesis process”, “nucleoside triphosphate biosynthesis process”, “cellular amino acid catabolism process”, etc. Its molecular function is related to the structural molecular activity and the structural composition of ribosomes. The KEGG pathway analysis revealed 72 pathways, including ribosomes, arginine biosynthesis, amino acid biosynthesis, 2-oxo-carboxylic acid metabolism, etc.

#### 3.3.3. Validation of DEGs Results by qRT-PCR Analysis

To validate the transcriptomic findings, eight randomly selected genes that showed significant differential expression were subjected to qRT-PCR. The qRT-PCR results (Figure S2) were highly consistent with the transcriptome data, thereby confirming the observed differential expression patterns. The consistency between qRT-PCR and transcriptome analysis confirms the robustness and reliability of our findings.

### 3.4. Metabolomic Results Analysis

**Figure 5:**
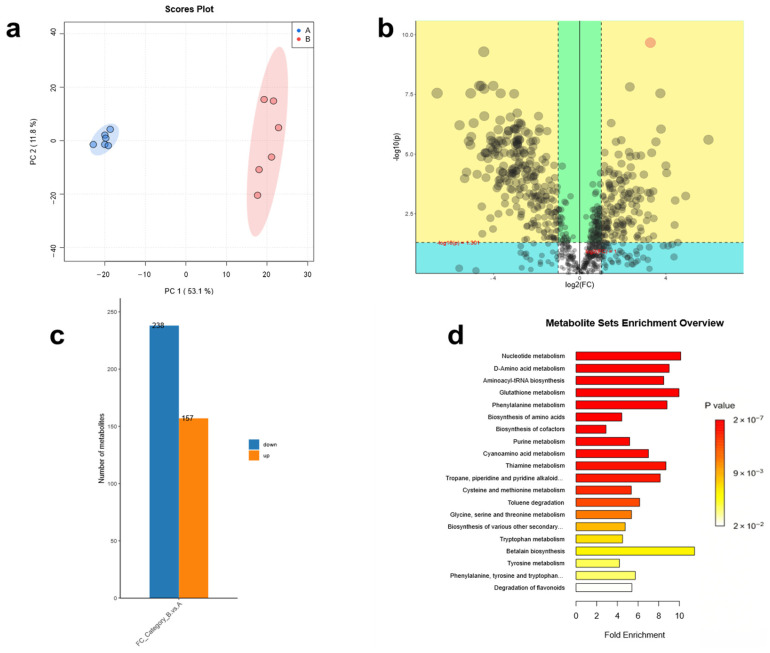
Untargeted LC-MS metabolomic profiling of MRSA after photocatalytic inactivation by 0.2 Ov-Bi_2_MoO_6_. (**a**) PCA scatter plot showing obvious metabolic phenotype separation between Group A (control) and Group B (treatment); (**b**) volcano plot for screening differentially abundant metabolites (DEMs); (**c**) statistical number of up-regulated and down-regulated DEMs; and (**d**) bubble chart of top enriched KEGG metabolic pathways based on differential metabolites.

Using LC-MS for untargeted metabolomics analysis of MRSA, PCA results (Figure 5a) indicated complete separation of control (Group A) and experimental group (Group B), indicating significant differences between the two groups. OPLS-DA (Figure S3) also showed that the metabolites of the two groups showed obvious separation, indicating that the OPLS-DA model based on the LC-MS analysis of MRSA metabolites could distinguish the metabolic profiles of bacteria under different treatment conditions. Based on the screening criteria of |log2 (fold change)| > 1 and *p* < 0.05, a total of 395 differentially expressed metabolites (DEMs) were screened compared with the control group (Group A) (Figure 5b). Among them, 157 DEMs were significantly up-regulated and 238 DEMs were significantly down-regulated (Figure 5c). Hierarchical clustering heatmap analysis showed that DEMs were also divided into two clusters (Figure S4). Through bioinformatics analysis, among 395 differential metabolites, a total of 152 substances were annotated into the KEGG database, which were involved in 81 metabolic pathways. As shown in the KEGG enrichment analysis diagram (Figure 5d), DEMs were enriched in nucleotide metabolism, biosynthesis of amino acids, aminoacyl-tRNA biosynthesis, glutathione metabolism, 2-oxo-carboxylic acid metabolism, and others. These results were partially consistent with those of RNA sequencing. Therefore, a joint analysis is necessary to further explore the correlation between transcriptomes and metabolomics.

### 3.5. Joint Analysis of Transcriptome and Metabolome

To systematically map the molecular effects of 0.2 Ov-Bi_2_MoO_6_ (OBM) against MRSA, we performed an association analysis on transcriptome and transcriptome data. This integrated analysis went beyond the single omics level to reveal the direct link between gene expression regulation and downstream target changes, and mapped the systemic response network of MRSA under photocatalytic stress.

#### 3.5.1. Validation of Synergism Between Pro-Activator and Protein Synthesis Block

The combined analysis confirmed that OBM treatment severely affected MRSA dependence. The regulome data showed that key genes involved in multiple regulator biosynthesis pathways were significantly inhibited, such as carA, carB and arcC in the arginine biosynthesis pathway, and aroA, pheA in tryptophan and phenylalanine biosynthesis, which were significantly regulated. As a result, transcriptomics detected a significant reduction in the intracellular abundance of various activated feedstocks such as arginine, proline, cysteine and tryptophan as end products. This chain reaction from transcription to the exhaustion of activating substances leads directly to protein synthesis. This conclusion is further supported by transcriptome data showing that multiple protein genes encoding 30S and 50S ribosomal subunits, such as rpsN, rpsS, and rplV, are expressed and transcribed, suggesting that the activity of the protein synthesis machinery is also inhibited.

#### 3.5.2. Energy-Induced Core Hub Locking

The integrative analysis clearly pointed to an energetic response crisis of MRSA in the OBM neighborhood. In the glycolytic/gluconeogenesis pathway, the down-regulation of key enzyme genes (such as gap, pgk, and pyk) despite the significantly reduced phase brightness in the presence of metabolite D-fructose-6-phosphate indicates pathway utilization efficiency stress. Some genes in the TCA cycle (e.g., sucC, sucD, and icd) showed compensatory up-regulation, but the content of the key intermediary help, cisaconitate, was significantly decreased, suggesting that the overall consumption of the TCA cycle may be blocked or the metabolic flow may be redistributed. Most critically, oxidative phosphorylation is central to cellular energy calling. The discovery of genes encoding subunits of the ATP synthase complex (atpA to atpH) and key components of the electron transport chain (e.g., cyoA, ctaA) in the adjustment group data revealed that the cessation of ATP synthesis is one of the most fundamental causes of bacterial inactivation.

#### 3.5.3. Mutual Corroboration of Nucleic Acid Metabolism Imbalance and Genetic Information Instability

The combined analysis revealed a severe imbalance of DNA replication and repair processes at both transcriptional and metabolic levels. Transcriptome data showed that genes critical for DNA replication initiation (dnaB, ssb) and mismatch repair genes (mutS, mutL) were down-regulated, indicating a block in DNA replication and decreased fidelity. Metabolomics provides a direct material basis for this: the content of pyrimidine metabolites, such as deoxycytidine, thymidine, and their monophosphate, which are used as raw materials for DNA synthesis, is significantly down-regulated, resulting in a serious shortage of synthetic raw materials. At the same time, the contents of adenine, guanine and xanthic acid in the purine metabolic pathway were abnormally up-regulated. This severe imbalance between purine and pyrimidine metabolites, which provides a direct metabolic explanation for the deranging of DNA replication and repair observed in the transcriptome, suggests that OBM interferes with the genetic stability of MRSA by disrupting the supply balance of nucleic acid precursor substances.

In summary, the combined transcriptome and metabolome analysis confirmed that the inactivation effect of 0.2 Ov-Bi_2_MoO_6_ on MRSA was not a single target action, but a multi-level and synergistic systemic attack. It chokes protein translation by inhibiting the synthesis of key amino acids, severs cellular energy supply by blocking oxidative phosphorylation, and interferes with the stability of genetic information by disrupting the balance of nucleotide metabolism, eventually leading to irreversible bacterial death.

## 4. Discussion

Bacteria sense signals and respond to environmental stresses by inducing the expression of relevant genes, and this response mechanism involves complex interactions between multiple genes, signaling pathways, and metabolic processes. Transcriptomics and metabolomics analysis can clarify the regulatory network at the molecular and metabolic levels, identify the key genes and metabolites involved in specific biological processes, and further study the relationship between regulation and feedback regulation between genes and metabolites, which provides a theoretical basis for studying the mechanism of oxygen-enriched vacancy bismuth molybdate photocatalytic inactivation of MRSA. The specific mechanism analysis is as follows:

(1) Disorders of amino acid metabolism and protein synthesis

In addition to building proteins in bacteria, amino acids also participate in important biological processes such as energy metabolism and signal transduction. Studies have shown that under the treatment of antibacterial agents, the metabolism and synthesis of amino acids in bacteria will be affected, resulting in disorder, thereby inhibiting the growth of bacteria. In transcriptome studies, genes involved in cysteine metabolism (cysK, mccA, luxS), tryptophan and phenylalanine biosynthesis (aroA, aroB, hisC, pheA, aroA), arginine biosynthesis (carB, carA, arcC, argF, ureC, ureA, ureB, purQ), proline metabolism (dat, putA, nos, proC), and isoleucine biosynthesis (tdcB, ilvE, alsS) were significantly down-regulated suggesting that amino acids may be in an imbalance in intracellular MRSA. Arginine is the basic unit of protein, and the biosynthesis of arginine can provide energy and maintain pH stability to sustain life activities. Under 0.2 Ov-Bi_2_MoO_6_ treatment, the biosynthesis of arginine was disrupted, arcC and ureC were significantly down-regulated, the synthesis of NH_3_ was inhibited, the content of OH^-^ was reduced, and the intracellular pH was maintained. “Significant down-regulation of carbamoyl phosphate synthase (carA, carbamoyl phosphate synthase small chain; carB, carbamoyl phosphate synthase large chain) and carbamoyl kinase (arcC) inhibits carbamoyl-P synthesis, leading to inhibition of the first step of the ornithine cycle, thereby disrupting arginine biosynthesis.” In the metabolomic study, the contents of cysteine, tryptophan, phenylalanine, arginine, proline, glutamine, and isoleucine were significantly down-regulated after treatment with 0.2 Ov-Bi_2_MoO_6_ (Schedule B), which coincided with the transcriptome results. These results suggest that 0.2 Ov-Bi_2_MoO_6_ can inhibit the metabolism and synthesis of MRSA-specific amino acids, which may lead to an imbalance in the intracellular pH and the reduction of intracellular nitrogen and carbon sources, ultimately hindering the growth of MRSA.

Proteins are synthesized in the ribosome using mRNA as a template, and protein synthesis requires amino acids as raw materials, and disorders in amino acid metabolism and synthesis may lead to disorders in protein synthesis. In transcriptome studies, genes encoding 16S ribosomal proteins (SABB_06260, SABB_06237, SABB_06060, SABB_06046, SABB_06283), 30S ribosomal proteins (rpsN, rpsS, rpsH), and 50S ribosomal proteins (rplV, rplX) were significantly down-regulated, suggesting that the intracellular protein translation capacity of MRSA may be inhibited. And the bases of 5S ribosomal protein (SABB_06051, SABB_06048, SABB_06281, SABB_06063) and 23S ribosomal protein (SABB_06047, SABB_06282, SABB_06259, SABB_06062, SABB_06234) Because of the significant up-regulation, it may be that MRSA is stressed by 0.2 Ov-Bi_2_MoO_6_, and the bacteria try to reduce cell damage by directed protein synthesis. Therefore, the reduction in the content of multiple amino acids in our study may also be attributed to the consumption of specific amino acids by protein synthesis.

(2) Damage to cell wall and membrane function

It was demonstrated in the study that the cell wall and membrane of the bacteria were damaged after 0.2 Ov-Bi_2_MoO_6_ treatment. In the transcriptome study, the genes of phospho-N-acetyl muramyl pentapeptidase (*mraY*) and alanine ligase (*ddl*, *murF*) were significantly down-regulated, suggesting that the peptidoglycan biosynthesis process may be inhibited, which in turn affects the integrity and stability of the bacterial cell wall.

**Figure 6:**
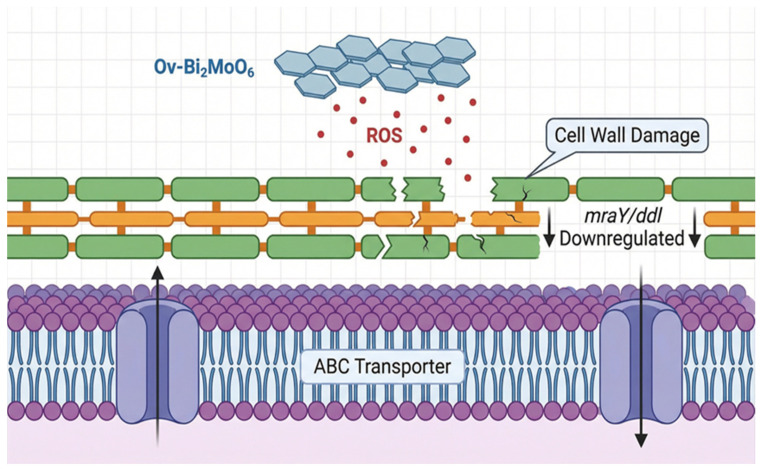
Mechanism schematic illustrating cell wall and membrane damage of MRSA triggered by 0.2 Ov-Bi_2_MoO_6_-derived reactive oxygen species (ROS). ROS attack peptidoglycan layer physically, while down-regulated expression of mraY and ddl genes further suppress cell wall biosynthesis; up-regulated ABC transporter-related genes reflect bacterial passive stress response after membrane structural injury.

The cell membrane is an important structural component of bacterial cells and plays a crucial role in maintaining cell morphology and energy metabolism. According to the results of the transcriptome analysis, five of the six DEGs related to galactose metabolism were up-regulated (*glk*, *pmmB*, *lacG*, *gat*, *cps2D*) and one was down-regulated (*yugT*). Products of galactose metabolism can be involved in the synthesis of glycoproteins and glycolipids on the cell membrane, which may be the cause of cell membrane damage. The cell internally wants to enhance galactose metabolism, synthesize more glycosylated components, and promote the repair of the cell membrane to reduce damage. D-fructose 6-phosphate content, which is associated with galactose metabolism, was significantly down-regulated in the metabolome study (Schedule B), which coincided with the transcriptomic findings. The ABC transport system is an important family of membrane proteins on the bacterial cell membrane, which is closely related to the transport of substances across the membrane. In transcriptome studies, ATP-binding proteins (glnQ, zurA, pstB, oppF) and permease proteins (arpJ, znuB, pstA) were significantly up-regulated, suggesting that the internal environment of the cell has been altered, and bacteria attempt to transport intracellular toxic substances outside the membrane by enhancing the function of the ABC transport system. In addition, nutrients are transported to the membrane in a reverse concentration to maintain the stability of the intracellular environment. These results suggest that the down-regulation of genes related to bacterial cell membrane biosynthesis leads to damage of the cell membrane structure, while the up-regulation of genes related to galactose metabolism and the ABC transport system may be an attempt by bacteria to repair the cell membrane and maintain the stability of the intracellular environment by exuding toxic and harmful substances and taking in nutrients to synthesize more glycosylation components. These changes suggest that bacteria regulate by multiple mechanisms in response to 0.2 OV-BI 2 moo6 damage to the cell wall and membrane (Figure 6).

The diagram shows the Ov—Bi_2_MoO_6_ nano slice through the release of reactive oxygen species (ROS) and direct contact with the peptidoglycan layer can cause a physical fracture. The down-regulation of key cell wall synthesis genes (*mraY* and *ddl*) further aggravated the cell wall damage. At the same time, the expression of ABC transporters on the cell membrane was up-regulated, reflecting the stress transport response of bacteria after membrane damage.

(3) Oxidative stress and energy metabolism disorder

As the fundamental stimulus–response coupling mechanism, the two-component system allows an organism to sense and respond to changes in many different environmental conditions. In the transcriptome study, genes involved in the nitrate reduction process were significantly down-regulated (*narH*, *narG*, *narJ*, *narK*) in the two-component system, suggesting that the bacteria may be in an oxidative stress environment, which in turn leads to the inhibition of the nitrate reduction pathway. The uhpT gene, which encodes a hexose phosphate transporter, is mainly involved in phosphate uptake, and its down-regulation may be related to changes in extracellular phosphate concentration. The significantly up-regulated lrgB gene is related to cell wall synthesis, which may be due to the enhanced repair or synthesis ability of the cell wall under stress, which is corresponding to the inhibition of peptidoglycan biosynthesis. The vraS gene is an important part of the two-component system, and its up-regulation may be responsible for the regulation of cell membrane integrity under stress conditions and enhance the effect of bacteria on environmental changes. Histidine reduces the damage caused by oxidative stress by regulating signaling pathways. In transcriptome studies, key genes in the histidine metabolism pathway (*hutG*, *hutU*, *hutI*, *hisE*, *hisF*, *hisA*, *hisH*, *hutH*) were significantly up-regulated, while the hisC gene was significantly down-regulated. This indicates that the histidine metabolic pathway is activated, which may be a stress response mechanism of bacteria under oxidative stress conditions. Urocanic acid and imidazolidone propionic acid are intermediates of hutG, hutU, hutI and hutH genes involved in histidine metabolism, which may be involved in the regulation of the intracellular REDOX state and reduce the damage caused by oxidative stress. The hisC gene encodes a histone phosphate aminotransferase, and its down-regulation may indicate that bacterial cells preferentially utilize exogenous histidine under oxidative stress conditions in response to oxidative stress. In the metabolomics study, the carnosine content related to histidine metabolism was significantly decreased, indicating that under oxidative stress, the intracellular antioxidant capacity was weakened, and the cells may try to enhance the antioxidant capacity against 0.2 Ov-Bi_2_MoO_6_ by up-regulating histidine metabolism-related genes. This was consistent with the transcriptome results.

Through substance metabolism, nutrients ingested by bacteria from the outside world go through glycolysis, the TCA cycle and the electron transport chain to provide energy for cell activities. In the transcriptome study, genes involved in glycolysis/glucose production (*pmmB*, *glk*, *pdhC*, *pdhB*, *bkdA1*, *lpdA*) were significantly up-regulated, indicating that bacteria may enhance the activity of metabolic pathways such as phosphoenolpyruvate (PEP) production or the oxidative decarboxylation process of pyruvate. However, *adhP*, *gap*, *pgk*, *gpmI*, *ptsG*, *gpmA*, *adhE*, *gapB*, *pyk*, *tpiA*, and *fda* genes were significantly down-regulated, indicating that the intracellular glycolysis pathway was inhibited, which may lead to decreased glucose uptake and metabolic efficiency. These changes in gene expression may reflect that bacteria preferentially utilize the pentose phosphate pathway to meet the energy and metabolic needs of cells under specific conditions, such as coping with oxidative stress or nutrient limitation. In the metabolome study, gluconic acid 6-phosphate, a metabolite related to the pentose phosphate pathway, was significantly up-regulated (Schedule B), while D-fructose 6-phosphate, which is related to starch and sucrose metabolism, and d-mannitol 1-phosphate, which is related to fructose and mannose metabolism, were significantly down-regulated (Schedule B). These results were consistent with the transcriptome results. It is suggested that bacteria may generate more NADPH and ribose phosphate by enhancing the pentose phosphate pathway to support the antioxidant defense and biosynthetic requirements of the cell. In transcriptome studies, genes involved in the TCA cycle (*sucD*, *sucC*, *sdhB*, *lpdA*, *pdhB*, *pdhC*, *icd*, *citZ*, *mqo*) were significantly up-regulated. Among these genes, *pdhC* and *pdhB*, which encode subunits of the pyruvate dehydrogenase complex, contribute to the conversion of pyruvate to acetyl-coa. Thus, upon entering the TCA cycle, up-regulation of the icd gene, encoding isocitrate dehydrogenase, contributes to increased production of NADH, which in turn generates more ATP through the electron transport chain. These results suggest that bacteria increase the efficiency of energy metabolism by enhancing the metabolic activity of the TCA cycle to provide more ATP for cellular activities. However, in metabolomic studies, cisaconitate, a metabolite associated with the TCA cycle, was significantly down-regulated (Schedule B), which may indicate that some branches of the TCA cycle are preferentially utilized, leading to reduced accumulation of some intermediate metabolites.

**Figure 7:**
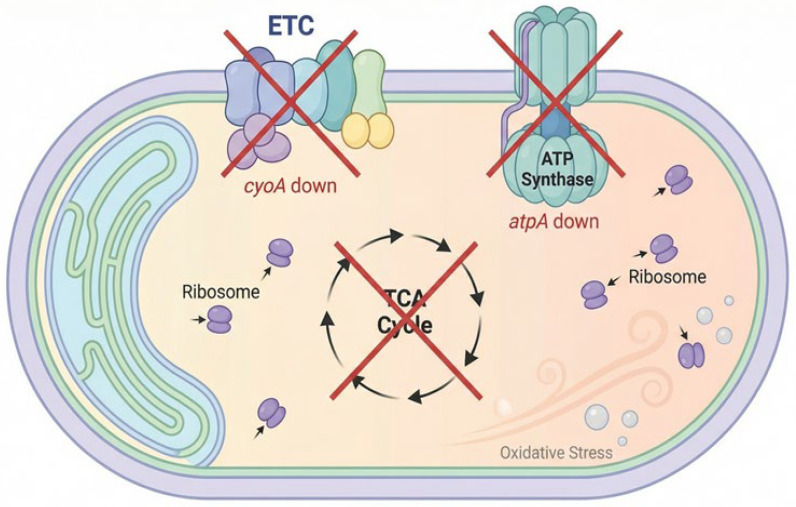
Schematic presentation of intracellular energy metabolism collapse in MRSA. Down-regulated cyoA (electron transport chain subunit) and atp family (ATP synthase) genes block electron transfer and ATP synthesis; suppressed TCA cycle pathway cuts off upstream energy precursor supply, eventually resulting in ribosome dysfunction and bacterial metabolic failure.

Riboflavin (vitamin B2) is an important coenzyme in cell metabolism and participates in a variety of REDOX reactions. In the transcriptome study, genes involved in riboflavin metabolism (*ABB_01508*, *ribH*, *ribE*, *ribD*, *nfrA*, *ribBA*) were significantly up-regulated, indicating that bacteria meet the cellular demand for energy metabolism and antioxidant defense by enhancing riboflavin synthesis capacity. The metabolites xanthic acid and 2, 6-dihydroxypurine, which are related to riboflavin metabolism, were significantly up-regulated in the metabolome study, which coincided with the transcriptome results. Oxidative phosphorylation is mainly responsible for electron transport, H+ transport and oxygen utilization in bacteria to produce H2O and ATP. In the transcriptome study, genes related to oxidative phosphorylation (*qoxB*, *atpC*, *atpD*, *atpG*, *atpA*, *atpH*, *atpF*, *atpE*, *atpB*, *cyoA*, *ctaA*) were significantly down-regulated. Among them, the down-regulation of the cyoA gene encoding NADH dehydrogenase, the ctaA gene encoding cytochrome oxidase, and the atpA and atpB genes encoding the ATP synthase subunit may affect the integrity of the electron transport chain and reduce the formation of proton kinetic potential, thereby inhibiting ATP synthesis The above results suggest that the down-regulation of genes involved in oxidative phosphorylation reflects the inhibition of bacterial energy metabolism, which may be a mechanism of action by 0.2 Ov-Bi_2_MoO_6_ to inactivate MRSA. C5 branched-chain dicarboxylic acid metabolism is an important pathway for energy metabolism and biosynthesis in organisms. In the transcriptome study, leuB, leuC and leuD genes encoding 3-isopropyl malate dehydrogenase were significantly up-regulated in C5 branched-chain dicarboxylic acid metabolism, indicating that bacteria enhanced the synthesis of leucine in C5 branched-chain dicarboxylic acid metabolism. The genes encoding succinyl-coa ligase, sucC and sucD, were significantly up-regulated, indicating that bacteria were enhancing the TCA cycle and optimizing C5 branched dicarboxylic acid metabolism at the same time. The budA gene, encoding α-acetyllactate decarboxylase, and the alsS gene, encoding acetyllactate synthase, were significantly down-regulated, indicating that the strain may reduce the production of metabolites such as acetoin and inhibit the initiation step of C5 branched-chain dicarboxylic acid metabolism. In metabolomic studies, cis-aconitate, a metabolite associated with C5 branched-chain dicarboxylic acid metabolism, was significantly down-regulated (Schedule B), possibly reflecting the inhibition of TCA cycle activity or redistribution of metabolic flux to other pathways, which coincided with the transcriptome results The above results suggest that 0.2 Ov-Bi_2_MoO_6_ may cause internal metabolic disorder by affecting the electron transport chain, reducing the efficiency of energy metabolism, and inhibiting the generation of ATP, which eventually leads to MRSA death (Figure 7).

This diagram depicts the central carbon metabolism pathway, systemic collapse. The red “X” mark shows the inhibition of the electron transport chain (ETC) and ATP synthase function, which is consistent with the significant down-regulation of the cyoA and atpA genes. Arrest of the TCA cycle (tricarboxylic acid cycle) cuts off the upstream pathway for energy production, leading to a dramatic drop in intracellular ATP levels and inactivation of ribosome function, ultimately triggering metabolic failure.

(4) Effect on nucleic acid

**Figure 8:**
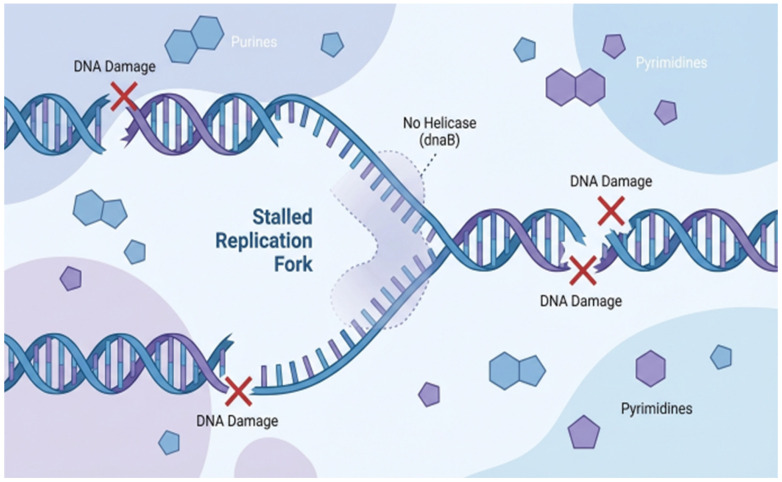
Schematic model of disrupted DNA replication and genomic instability in MRSA. Down-regulation of dnaB (DNA helicase) and ssb hinders the initiation of DNA replication; unbalanced purine–pyrimidine pool derived from disturbed nucleotide metabolism aggravates replication fork stagnation and DNA double-strand break accumulation.

As a carrier of genetic information, nucleic acids are essential biological macromolecules for life. In the transcriptome study, the genes *dnaG*, *holA*, *polC* and *dnaN* involved in the elongation and proofreading process of DNA replication were significantly up-regulated, indicating that bacteria may accelerate DNA replication or repair damaged DNA. However, dnaB and ssb genes were significantly down-regulated, among which the dnaB gene encodes helicase, a key enzyme in the initiation of DNA replication, and the ssb gene encodes a single-stranded binding protein that can protect single-stranded DNA from degradation. The down-regulation of these genes may lead to the obstruction of DNA replication initiation, which in turn induces replication disorders, which may be a mechanism by which 0.2 Ov-Bi_2_MoO_6_ inhibits MRSA activity. The genes related to RNA degradation, groL and dnaK, were significantly up-regulated, which may protect the activity of related enzymes during RNA degradation. However, the pnp gene was significantly down-regulated, which may lead to a lower efficiency of RNA degradation, and the above results indicate the regulation of RNA stability by bacteria under 0.2 OV-BI 2 moo6 treatment. The genes *recA*, *recO*, *recF*, and *recD2* that are associated with homologous recombination were significantly up-regulated, indicating that bacteria may be enhancing the homologous recombination mechanism, which may be caused by double-strand breaks or other severe damage during DNA replication caused by 0.2 Ov-Bi_2_MoO_6_. In the process of base excision repair, mpg was significantly up-regulated and nth was significantly down-regulated, indicating that bacteria may enhance the repair ability of certain damage types and weaken the repair ability of other damage types. The down-regulation of mismatch repair genes may lead to the accumulation of errors during DNA replication and further hinder the normal progress of replication. This leads to disorders in the body. Genes related to mismatch repair, *xseB*, *mutS*, and *mutL*, were significantly down-regulated, indicating that the activity of the mismatch repair pathway may be inhibited, resulting in decreased accuracy of DNA replication and an increased risk of mutations. In the metabolome study, the metabolites 2, 6-dihydroxypurine, adenosine diphosphate (ADP), 5′-adenylate, xanthic acid, adenine, and guanine, which are related to purine metabolism, were significantly up-regulated, and deoxyadenylate and hypoxanthine were significantly decreased. The metabolites deoxycytidine, 2-deoxyuridine, thymidine 5′-monophosphate, and thymidine, which are associated with pyrimidine metabolism, were significantly down-regulated. The up-regulation of key metabolites in purine metabolism and the down-regulation of key metabolites in pyrimidine metabolism suggest that an imbalance between purine and pyrimidine nucleotide supply may occur during DNA replication in bacteria. While DNA replication requires sufficient purine and pyrimidine nucleotides as raw materials, changes in metabolite levels may reflect metabolic bottlenecks encountered by bacteria during replication. Bacteria may sacrifice the efficiency of DNA replication by adjusting purine and pyrimidine metabolic pathways to preferentially meet other metabolic demands. The enhanced purine metabolism may be related to the cellular response to oxidative stress or other insults, such as the role of xanthine oxidase in the production of superoxide radicals. The changes in purine and pyrimidine metabolites in the metabolomic studies revealed metabolic disorders that bacteria may face during DNA replication, which coincided with the transcriptome results. The above results indicated that when bacteria were treated with 0.2 Ov-Bi_2_MoO_6_, DNA replication would be hindered and then disordered, RNA degradation efficiency would be reduced, homologous recombination would be disordered, and base excision repair errors would be accumulated, leading to metabolism and synthesis disorder in MRSA cells (Figure 8).

The diagram shows the fatal defects in DNA replication. Replication proceeds normally in a stalled replication fork due to the deletion of the helicase gene (*dnaB*) (indicated by the dashed line in the figure). The imbalance between purine and pyrimidine pools in the background further aggravates replication stress, which eventually leads to DNA double-strand breaks (DSBs) and severe genomic damage.

In this study, the multi-target molecular mechanism of oxygen-enriched vacancy bismuth molybdoate (0.2 Ov-Bi_2_MoO_6_) photocatalytic inactivation of MRSA was systematically analyzed for the first time through combined transcriptomics and metabolomics analysis. It was confirmed that the antibacterial effect of the photocatalytic antibacterial materials could be achieved through multiple pathways, such as disrupting amino acid metabolism, destroying the structure of the cell wall and cell membrane, inducing oxidative stress and energy metabolism disorders, and hindering the repair of nucleic acid replication. Compared with the current photocatalytic antibacterial materials, the photocatalytic antibacterial materials showed significant advantages of multiple targets and broad pathways, and did not easily cause bacterial resistance. Crucially, by integrating multi-omics, our study distinguishes the primary molecular targets of 0.2 Ov-Bi_2_MoO_6_ from secondary downstream effects, establishing a clear causality rather than mere correlation. Given the short half-life of ROS, the primary targets are inevitably the external cellular envelopes, causing initial physical oxidation and structural damage. Subsequently, the destruction of membrane integrity forces the bacteria into a severe stress state, triggering a cascade of secondary downstream metabolic exhaustions (such as amino acid depletion and DNA synthesis blockage), ultimately leading to irreversible cell death.

Reactive oxygen species (ROS) produced by photocatalytic materials is the core initial factor of this antibacterial action. In this study, the 0.2 Ov-Bi_2_MoO_6_ photocatalytic system continuously generated •O^2−^, •OH, ^1^O_2_ and other ROS, which caused a significant increase in the lipid peroxidation level of MRSA. This is consistent with the oxidative stress characteristics of oxygen-vacancy TiO_2_ photocatalytic materials prepared in previous studies when they inactivate *E. coli* [21], and also with the time-dependent increase in MDA content in defect-rich BioX-based materials reported in previous studies [22]. It is worth noting that although the SOD and Gsh-px activities of MRSA in this study were significantly up-regulated with the accumulation of ROS, they could not offset oxidative damage, which was consistent with the conclusion that the antioxidant enzyme system of drug-resistant bacteria was activated in a compensatory manner but failed under the stress of high concentrations of ROS [23]. These results suggest that the ROS flux generated by the 0.2 Ov-Bi_2_MoO_6_ photocatalytic system has exceeded the antioxidant defense threshold of MRSA, which is one of the key reasons for the high efficiency of the 0.2 Ov-Bi_2_MoO_6_ photocatalytic system in inactivating drug-resistant bacteria. Although direct physicochemical quantifications of ROS and band structures (such as EPR spectroscopy and Mott–Schottky plots) are classical approaches in materials science, this study distinctively leverages the robust multi-omics data as direct in vivo biological evidence of continuous ROS generation. The successful introduction of oxygen vacancies creates defect states that satisfy the thermodynamic requirements for sustained ROS production under visible light, directly triggering the subsequent biological cascade described above. The introduction of oxygen vacancy is the core modification strategy to improve the photocatalytic activity of Bi_2_MoO_6_. In this study, the oxygen vacancy content of 0.2 Ov-Bi_2_MoO_6_ was optimized to achieve efficient and sustained release of ROS. This is consistent with the theory that oxygen vacancies can be used as the capture site of photogenerated electron–hole pairs and inhibit their recombination, as proposed by previous studies [24], and also provides an experimental basis for the subsequent defect engineering modification of bismuth-based photocatalytic materials.

Amino acid metabolism disorder is an important molecular target of 0.2 Ov-Bi_2_MoO_6_ to inhibit the growth of MRSA. In this study, we found that the synthesis genes of arginine, phenylalanine, cysteine and other amino acids were significantly down-regulated and the content of metabolites was decreased. This is similar to the feature of extensive inhibition of amino acid biosynthesis pathways found in previous studies using TiO_2_-Ag-AgCl composites to inactivate Escherichia coli [25]. Arginine metabolism is the most significantly affected amino acid metabolism pathway in this study, and the down-regulation of its key genes arcC and carB leads to a block of the ornithine cycle, which is consistent with the mechanism of intracellular pH imbalance caused by disordered arginine metabolism when antibacterial peptides act on MRSA reported in previous studies [26]. In addition, the disorder of amino acid metabolism in this study further leads to the differential expression of ribosomal protein genes, leading to disorder of the protein synthesis system. This one-stage cascade reaction is highly consistent with previous studies indicating that carbon-spot photocatalytic materials block bacterial protein translation by inhibiting amino acid metabolism [27]. It was confirmed that the amino acid metabolism–protein synthesis axis was the universal target of photocatalytic antibacterial materials, and 0.2 Ov-Bi_2_MoO_6_ was specific and efficient to regulate this target.

The structural damage of the cell wall and cell membrane is an important link in the inactivation of MRSA by 0.2 Ov-Bi_2_MoO_6_. In this study, the down-regulation of the key peptidoglycan synthesis genes mraY and ddl resulted in the damage of cell wall integrity, which was consistent with the mechanism of cell wall damage when MoS_2_/Bi_2_MoO_6_ heterojunction materials prepared in previous studies were antibacterial [28]. After cell membrane damage, MRSA compensates for repair by up-regulating genes related to galactose metabolism and the ABC transport system. This stress response is similar to the repair mechanism of the cell membrane of *Staphylococcus aureus* treated with copper-based photocatalytic materials in previous studies [29], but the repair fails due to excessive damage. These results suggest that 0.2 Ov-Bi_2_MoO_6_ damages the bacterial membrane structure irreversibly. It is worth noting that the up-regulation of ABC transport system-related genes in this study did not lead to the effective discharge of intracellular toxic substances, which is consistent with the conclusion that the membrane transport system function of drug-resistant bacteria is impaired under photocatalytic ROS stress, as reported in previous studies [30], further confirming that 0.2 Ov-Bi_2_MoO_6_ can damage bacterial membrane function through multiple pathways, rather than a single physical injury.

Energy metabolism disorder is the core lethal mechanism of 0.2 Ov-Bi_2_MoO_6_ leading to MRSA death. This study found that the material can inhibit glycolysis, oxidative phosphorylation and other core energy metabolism pathways at the same time, which is consistent with the action mode of g-C_3_N_4_-based photocatalytic material reported by previous studies to inactivate drug-resistant bacteria by blocking energy metabolism [31]. Among them, the significant down-regulation of oxidative phosphorylation-related genes atpA, atpB, and cyoA leads to the blockage of ATP synthesis, which is consistent with the mechanism of oxygen-vacancy Bi_2_WO_6_ photocatalytic materials inhibiting the electron transport chain of Escherichia coli, as found in previous studies [32]. However, MRSA compensates for energy by up-regulating genes related to the TCA cycle and pentose phosphate pathway, but the compensation fails due to the imbalance of the metabolic flux of key metabolites, including cisaconionic acid and 6-phosphogluconic acid. This phenomenon is consistent with the theory that photocatalytic ROS can cause metabolic reprogramming disorder of the bacterial energy metabolism pathway proposed by previous studies [33]. In addition, the up-regulation of genes related to riboflavin metabolism in this study is an important stress response of bacteria to cope with energy metabolism disorders. Riboflavin is a key coenzyme in REDOX reactions, and its synthesis is enhanced to try to alleviate the dysfunction of the electron transport chain, which is consistent with the compensatory activation of riboflavin metabolism in bacteria under energy stress, as found in previous studies [34]. It is also confirmed that 0.2 Ov-Bi_2_MoO_6_ results in comprehensive and irreversible inhibition of MRSA energy metabolism.

The disorder of nucleic acid replication and repair system is an important subsequent effect of 0.2 Ov-Bi_2_MoO_6_ inhibiting MRSA proliferation and causing cell death. In this study, the down-regulation of DNA replication initiation key genes dnaB and ssb and the up-regulation of extended proofreading genes *dnaG* and *polC* resulted in disorder of the DNA replication process. This is similar to the mechanism of nucleic acid damage in the inactivation of MRSA by the graphene quantum dot photocatalytic materials reported in previous studies [35]. However, the down-regulation of mismatch repair genes mutS and mutL leads to the accumulation of DNA replication errors, while the up-regulation of homologous recombination genes *recA* and *recO* fails to achieve effective repair. The functional imbalance of this repair system is consistent with the conclusion that oxygen-rich vacancy ZnO photocatalytic materials cause bacterial DNA double-strand breaks and repair failure [36]. Metabolomics results showed that the supply of purine and pyrimidine metabolites was unbalanced, which further aggravated the metabolic bottleneck of nucleic acid replication; it was confirmed by previous studies that photocatalytic ROS could inhibit bacterial nucleic acid synthesis by disturbing nucleotide metabolism [37]. Unlike traditional antibacterial drugs, 0.2 Ov-Bi_2_MoO_6_ can affect multiple links of DNA replication, repair and nucleotide synthesis at the same time, which greatly reduces the possibility of bacterial resistance through a single gene mutation.

The results of this study also present two significant innovations compared with existing studies on photocatalytic antibacterial mechanisms: Firstly, it is confirmed for the first time that bismuth-based photocatalytic materials can achieve efficient inactivation of MRSA through multi-target regulation at the transcriptional and metabolic levels, breaking through the research limitations of bismuth-based materials that only demonstrate antibacterial activity at the cellular level and the lack of a molecular mechanism analysis in previous studies [38]. Secondly, it was found that the introduction of oxygen vacancies can expand the antibacterial targets of Bi_2_MoO_6_ from traditional membrane damage and oxidative stress to multiple pathways such as amino acid metabolism, energy metabolism, and nucleic acid metabolism, which provides a new molecular basis for defect engineering modification to improve the antibacterial activity and broad spectrum of photocatalytic materials [39]. In addition, the combined analysis strategy of transcriptomics and metabolomics used in this study makes up for the deficiency that exists because single omics technology cannot analyze gene–metabolite regulatory networks, which is consistent with the view that multi-omics technology is the optimal strategy to analyze the molecular mechanism of photocatalytic antibacterial effects [40]. It also provides a methodological reference for subsequent research on the antibacterial mechanism of other photocatalytic materials.

The essential difference between the multi-target photocatalytic killing and the traditional antibiotic antibacterial mode: The antibacterial effect of traditional antibiotics (such as vancomycin and methicillin) usually relies on the “lock and key effect”, that is, by specifically binding to a single target in the bacteria to inhibit bacterial growth, which is very likely to induce bacterial resistance. In contrast, this study found that the photocatalytic process mediated by 0.2 Ov-Bi_2_MoO_6_ exhibits a unique “systemic killing” feature: (1) The leap from inhibition to killing: Traditional antibiotics often show only temporary inhibitory effects at sub-lethal concentrations, while photocatalysis triggers irreversible lipid peroxidation and protein denaturation, directly leading to the disintegration of the bacterial structure. (2) Multi-site coordinated attack: Omics data show that OBM not only disrupts membrane integrity but also simultaneously shuts down three core systems at the molecular level: energy supply (ATP synthesis), raw material provision (amino acid synthesis), and genetic repair (down-regulation of *dnaB*). (3) Extremely low risk of resistance: This “all-round attack” makes it difficult for bacteria to survive through mutations in a single pathway, fundamentally distinguishing it from the limitations of traditional antibiotics.

From the perspective of application, the multi-target molecular mechanism of the 0.2 Ov-Bi_2_MoO_6_ photocatalytic inactivation of MRSA makes it a potential alternative to traditional antibiotics for the prevention and control of MRSA infection. Traditional antibiotics mostly act through a single target, such as β-lactams on cell wall synthesis and quinolones on nucleic acid replication, which easily lead to bacterial resistance through gene mutations or horizontal transfer [41]. However, the multi-target action mode of 0.2 Ov-Bi_2_MoO_6_ greatly reduces the probability of bacterial resistance. This advantage is also confirmed by the fact that MRSA did not develop resistance to the material after multiple passages in the present study. At the same time, 0.2 Ov-Bi_2_MoO_6_ can exert antibacterial activity under visible light, and the material itself has no obvious cytotoxicity, which is consistent with the biocompatibility characteristics of oxygen-vacancy bismuth-based materials reported in previous studies [42], making it a broad application prospect in the fields of surface modification of biomedical materials and control of MRSA pollution in the environment.

This study also has certain limitations, and subsequent studies can be further studied from the following aspects: first, this study only analyzed the antibacterial molecular mechanism of 0.2 Ov-Bi_2_MoO_6_ in vitro, and subsequently constructed an in vivo MRSA infection model to verify the antibacterial effect and mechanism of the material in vivo. Secondly, the function of the key differential genes (such as *arcC*, *dnaB*, *atpA*) in the 0.2 Ov-Bi_2_MoO_6_ antibacterial process can be verified by gene knockout and overexpression technology. Thirdly, the morphology and oxygen vacancy content of 0.2 Ov-Bi_2_MoO_6_ can be further optimized. Combined with the molecular mechanism results of this study, bismuth-based photocatalytic materials with stronger targeting and a higher antibacterial efficiency can be designed. Fourthly, for practical clinical and environmental applications, the long-term cyclic stability and reusability of the Ov-Bi_2_MoO_6_ catalyst under standard treatment conditions needs to be systematically evaluated in future studies.

In summary, the molecular mechanism of 0.2 Ov-Bi_2_MoO_6_ photocatalytic inactivation of MRSA analyzed by multi-omics technology in this study not only provides a theoretical basis for antibacterial research of bismuth-based photocatalytic materials, but also provides new targets and design ideas for the development of new anti-MRSA photocatalytic materials. At the same time, it provides a new strategy and method to solve the clinical MRSA resistance problem.

## 5. Conclusions

In this study, we demonstrated for the first time the photocatalytic inactivation mechanism of oxygen-rich vacancy bismuth molybdoate (0.2 Ov-Bi_2_MoO_6_) in methicillin-resistant *Staphylococcus aureus* (MRSA) through the combination of transcriptomics and metabolomics. Multi-omics analysis showed that 0.2 Ov-Bi_2_MoO_6_ caused irreversible death of MRSA through a multi-pathway and multi-level coordinated lethal attack. The key findings were as follows:

0.2 Ov-Bi_2_MoO_6_ inhibits bacterial stress repair by disrupting the protein synthesis system. This material not only significantly inhibited the biosynthetic pathways of arginine, cysteine, tryptophan and other key amino acids, but also cut off the raw material supply for protein synthesis. It also downregulates the expression of genes encoding key subunits of the ribosome, disrupting the core machinery of protein synthesis, and eventually leading to the complete arrest of protein translation activity.

0.2 Ov-Bi_2_MoO_6_ disrupts the core life activities of bacteria by triggering a catastrophic collapse of energy metabolism. This material severely inhibited the glycolytic pathway and destroyed the function of key nodes in the oxidative phosphorylation electron transport chain, such as NADH dehydrogenase and cytochrome oxidase, with ATP synthase, thereby fundamentally blocking ATP production and throwing MRSA into a lethal energy crisis.

0.2 Ov-Bi_2_MoO_6_ can inhibit bacterial proliferation and survival by disrupting the homeostasis and fidelity of genetic material. This material causes a serious imbalance between purine and pyrimidine metabolism, impedes the initiation of DNA replication (down-regulating gyrase dnaB and single-stranded binding protein ssb), and impairs the function of the DNA mismatch repair system, ultimately leading to DNA replication disorders and mutation accumulation.

In conclusion, this study has reached a comparative conclusion that differs from traditional antibacterial research: Unlike the selective inhibition of a single biological enzyme by traditional antibiotics, 0.2 Ov-Bi_2_MoO_6_ photocatalytic inactivation is characterized by a “broad-spectrum oxidative stress-mediated systemic collapse”. MRSA does not die from the closure of a single metabolic pathway, but from the combined effects of energy depletion and genetic instability. Compared with known photocatalytic materials, the introduction of oxygen vacancies significantly enhances the ROS flux, making its antibacterial efficiency surpass that of conventional defect-free materials. This multi-target, low-resistance-risk inactivation strategy provides important molecular biological support for the application of perovskite-like photocatalytic materials in the biomedical field.

## Data Availability

The raw transcriptome sequencing data generated in this study are not publicly available due to ongoing analyses but can be obtained from the corresponding author upon reasonable request. The metabolomics raw data and all other data supporting the findings of this study are available from the corresponding author upon reasonable request.

## Supplementary Materials

The following supporting information can be downloaded at: https://www.mdpi.com/article/10.3390/biology15130993/s1, Table S1: The genes and gene-specific primers used for qRT-PCR; Table S2: Statistics of transcriptome sequencing data; Table S3: Statistics of the mapping sequencing data to the reference genome; Figure S1: Correlation analysis of transcriptome samples and statistical distribution of differentially expressed genes (DEGs); Figure S2: qRT-PCR validation of selected differentially expressed genes consistent with RNA-seq data; Figure S3: OPLS-DA discriminant analysis of metabolome data for Group A and Group B; Figure S4: Hierarchical clustering heatmap of all differential metabolites; Figure S5: XRD, FT-IR, EPR, and XPS spectra analysis; Figure S6: UV–visible diffuse reflectance spectra, bandgap, photocurrent density and Nyquist impedance plots; Figure S7: Mott-Schottky diagram of Bi_2_MoO_6_.

## Abbreviations

The following abbreviations are used in this manuscript:

MRSA	Methicillin-resistant *Staphylococcus aureus*
OBM	Ov-Bi_2_MoO_6_
