# Supplementary material for: Transcriptomic and Metabolomic Analysis Reveals Molecular Mechanism of Oxygen-Rich Vacancy Bi2MoO6 Photocatalytic Inactivation of MRSA
Source: Biology (Basel). 2026 Jun 24;15(13):993. doi: 10.3390/biology15130993 (PMC13359974; doi:10.3390/biology15130993)
Supplement: Supplementary file 1 [file biology-15-00993-s001.zip › biology-4342407-supplementary.pdf]

Support Information

Table S1 The genes and gene-specific primers used for qRT-PCR.

Gene ID	Gene name	NR annotation	Primer sequence (5'-3')
Zosma01g26400	*fpp6*	Filament-like plant protein 6	F: AATTCTCCGAAGATGTGCTCAG<br>R: CTCGCTCCTCTGGAAGTTGA
Zosma360g00160	*nd4*	NADH dehydrogenase subunit 4	F: CGGTCTGCCATAGTCCTACT<br>R: GTGAATCGGTGGTTCCTGTT
Zosma70g00060	*psbb*	Photosystem II CP47 reaction center protein	F: TAGTGTCCGTCCGCCTCAA<br>R: ACCACATAGTTCCAGCAACAAC
Zosma70g00260	*psba*	Photosystem II protein D1	F: GGTATGCGTCCTTGGATTGC<br>R: GGATATTGTGTTCTGCCTGGAA
Zosma01g36210	*wsd1*	Wax ester synthase/diacylglycerol acyltransferase 1	F: TCACATACATTCGCTCCACATC<br>R: ATCGCATTACGCATTCCTTCAT
Zosma05g14170	*xi-e*	Myosin XI	F: TGGTATCGGAGCAGTTCAAGG<br>R: GGTCTCGGCAACAACATCAAC
Zosma05g25200	*hppr*	hydroxyphenylpyruvate reductase	F: AAGGTTCACGCCGATTCCA<br>R: ACATCCTCCGTCAACACATCA
Zosma03g29870	*snap25*	Synaptosomal-associated protein 25	F: GGTCATCTTCCGAACCATCTCT<br>R: TTGTAGTGTCTTCCGCCTTGT
Zosma03g18260	*tbl26*	Trichome birefringence-like 26	F: ACCGCCTTGTAACTATGATCTG<br>R: TTGTCATCTGCTTCAACGAGAA

Table S2 Statistics of transcriptome sequencing data.

Sample	Raw reads	Clean reads	Q20(%)	Q30(%)	GC content(%)
A1	14003350	13959050	99.20	97.31	34.32
A2	16085890	16043144	99.20	97.31	35.98
A3	13572062	13527172	99.22	97.39	34.65
A4	15379976	15335306	99.23	97.44	34.26
A5	15677152	15630844	99.26	97.51	34.37
A6	14076096	14043522	99.21	97.35	35.57
B1	20537452	20479878	99.20	97.32	34.49
B2	15650066	15530178	99.19	97.28	36.63
B3	13352154	13307406	99.20	97.31	35.35
B4	14038528	13999220	99.26	97.54	35.17
B5	20997624	20891468	99.27	97.56	36.11
B6	15031358	14987206	99.26	97.50	34.71

Table S3 Statistics of the mapping sequencing data to the reference genome.

Sample	Total mapped	Multiple mapped	Uniquely mapped
A1	13585330(97.32%)	74022(0.53%)	13511308(96.79%)
A2	15666362(97.65%)	1629756(10.16%)	14036606(87.49%)
A3	13100901(96.85%)	415575(3.07%)	12685326(93.78%)
A4	14883637(97.05%)	93895(0.61%)	14789742(96.44%)
A5	15051243(96.29%)	168800(1.08%)	14882443(95.21%)
A6	13629823(97.05%)	1155102(8.23%)	12474721(88.82%)
B1	19901743(97.18%)	163776(0.8%)	19737967(96.38%)
B2	14935655(96.17%)	2603275(16.76%)	12332380(79.41%)
B3	12923273(97.11%)	916362(6.89%)	12006911(90.22%)

B4	13587912(97.06%)	857815(6.13%)	12730097(90.93%)
B5	20070044(96.07%)	2403346(11.5%)	17666698(84.57%)
B6	14550865(97.09%)	210415(1.4%)	14340450(95.69%)

Figure S1

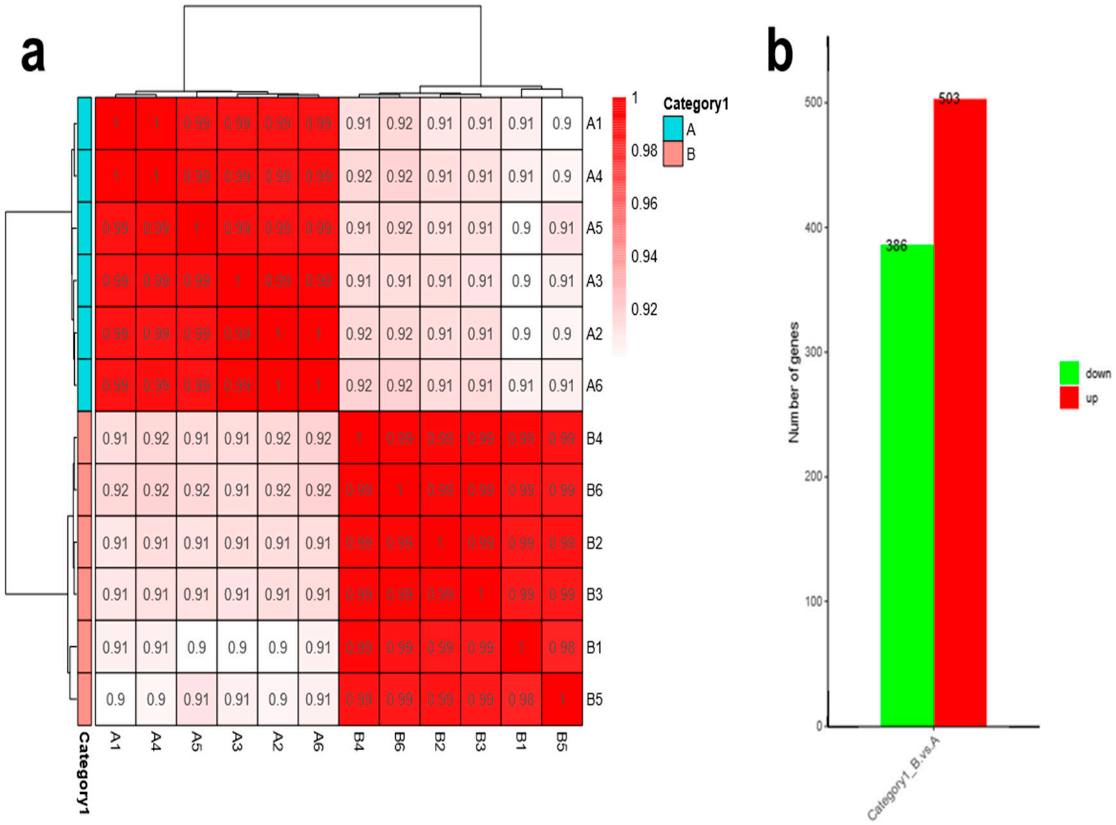

Figure S1. Correlation analysis of transcriptome samples and statistical distribution of differentially expressed genes (DEGs). (a) Pearson correlation heatmap of gene expression profiles across all biological replicates from control group (A1－A6, cyan) and Ov-Bi_2MoO_6-treated group (B1–B6, salmon red). The numerical values in the heatmap represent pairwise correlation coefficients between different samples; high correlation values (>0.90) demonstrate favorable intra-group repeatability and obvious inter-group expression divergence. (b) Bar statistics for the number of significantly differentially expressed genes between treated and control groups, in which green bars correspond to down-regulated genes (386 genes) and red bars represent up-regulated genes (503 genes).

Figure S2

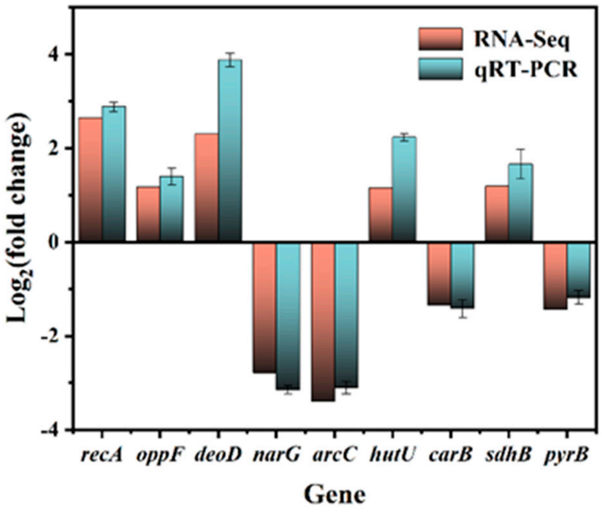

Figure S2. qRT-PCR validation of selected differentially expressed genes consistent with RNA-seq data. Relative expression fold change of nine randomly selected DEGs quantified by qRT-PCR; the consistent expression tendency between qRT-PCR and transcriptome sequencing verifies the reliability of RNA-seq results.

Figure S3

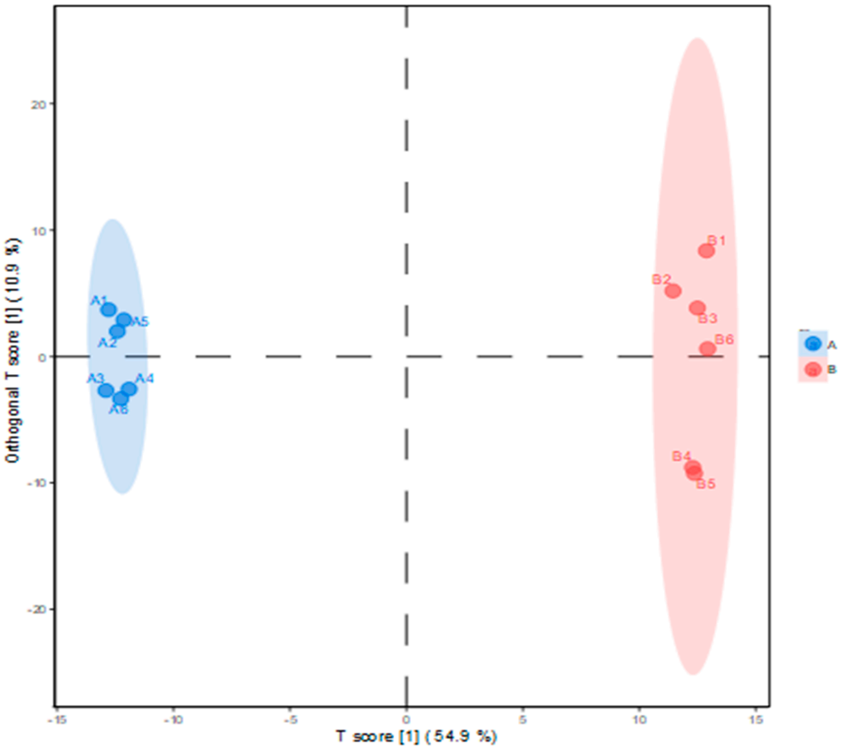

Figure S3. OPLS-DA discriminant analysis of metabolome data for Group A and Group B. OPLS-DA score plot based on LC-MS metabolomics data, demonstrating complete metabolic profile discrimination between blank control (A) and 0.2 Ov-Bi_2MoO_6 treated MRSA (B).

Figure S4

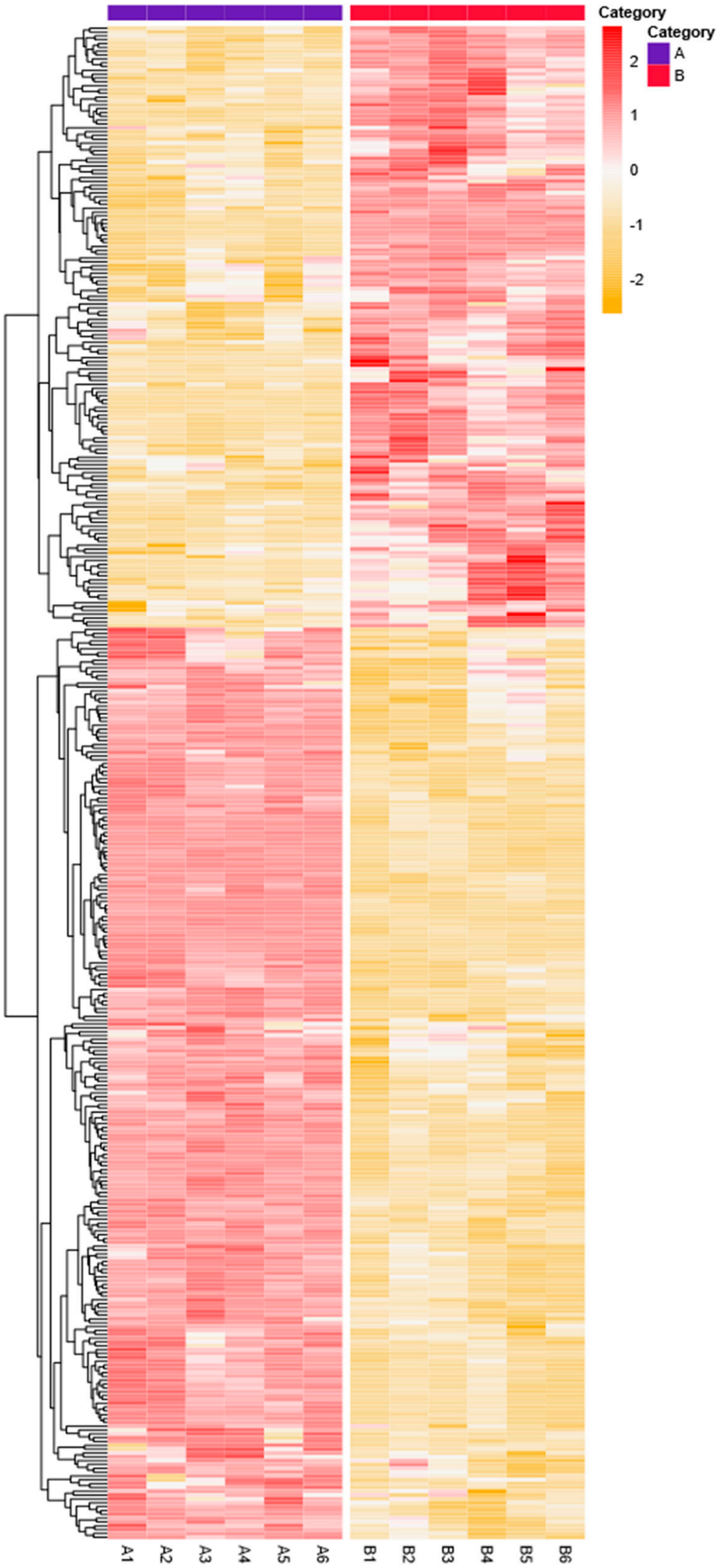

Figure S4. Hierarchical clustering heatmap of all differential metabolites. Figure S4. Cluster heatmap of differentially abundant metabolites (DEMs) from MRSA samples; samples are clearly clustered into two independent branches corresponding to Group A and Group B.

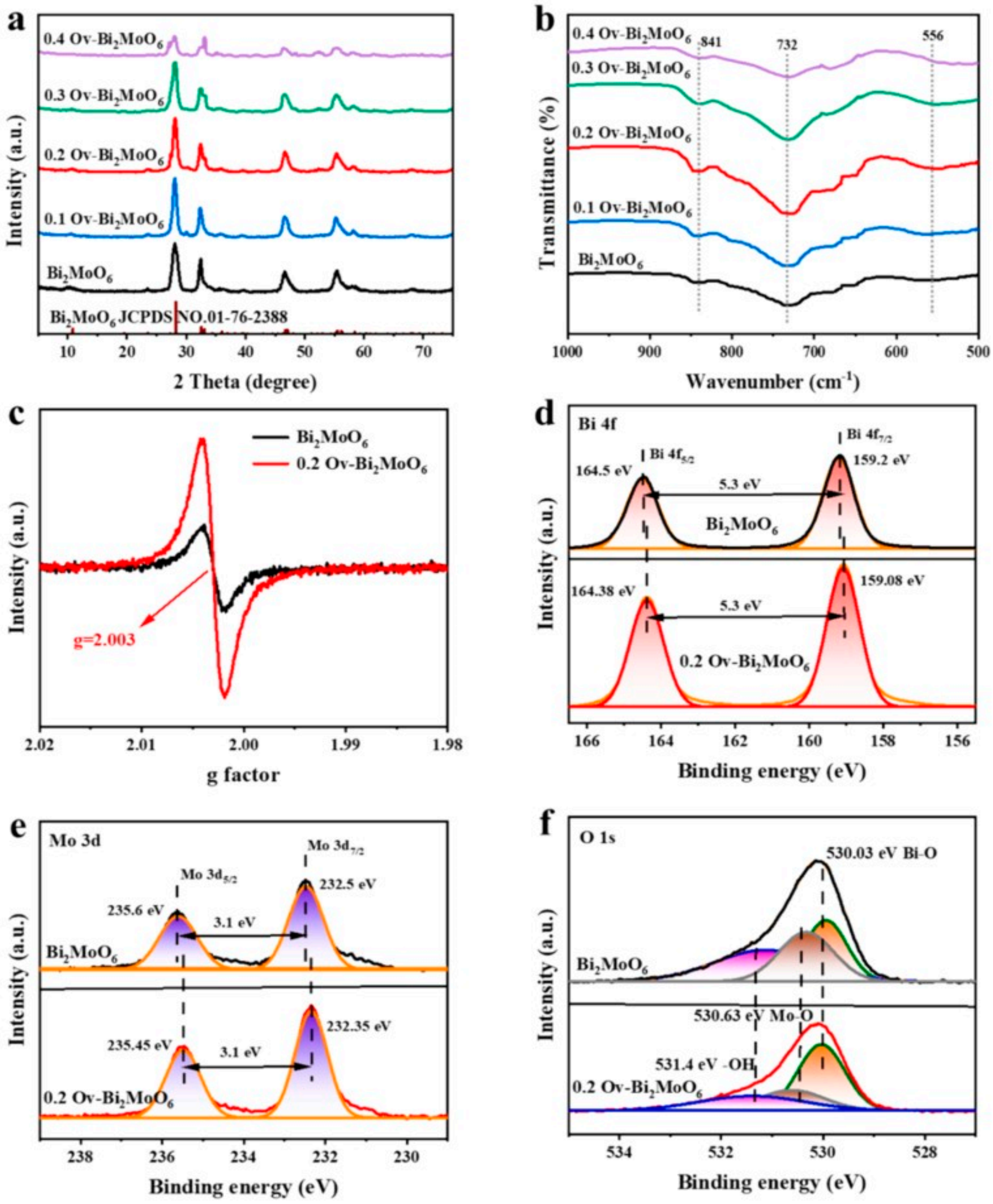

Figure S5. (a) XRD spectra of Bi2MoO6 and four Ov-Bi2MoO6 samples; (b) FT-IR spectra for Bi2MoO6 and four Ov-Bi2MoO6 samples with range from 500 to 1000 cm- 1; (c) EPR spectra of Bi2MoO6 and four Ov-Bi2MoO6 samples; (d) The high-resolution Bi 4f XPS spectra of Bi2MoO6 and 0.2 Ov-Bi2MoO6; (e) The high-resolution Mo 3d XPS spectra of Bi2MoO6 and 0.2 Ov-Bi2MoO6; (f) The high-resolution O 1s XPS spectra of Bi2MoO6 and 0.2 Ov-Bi2MoO6.

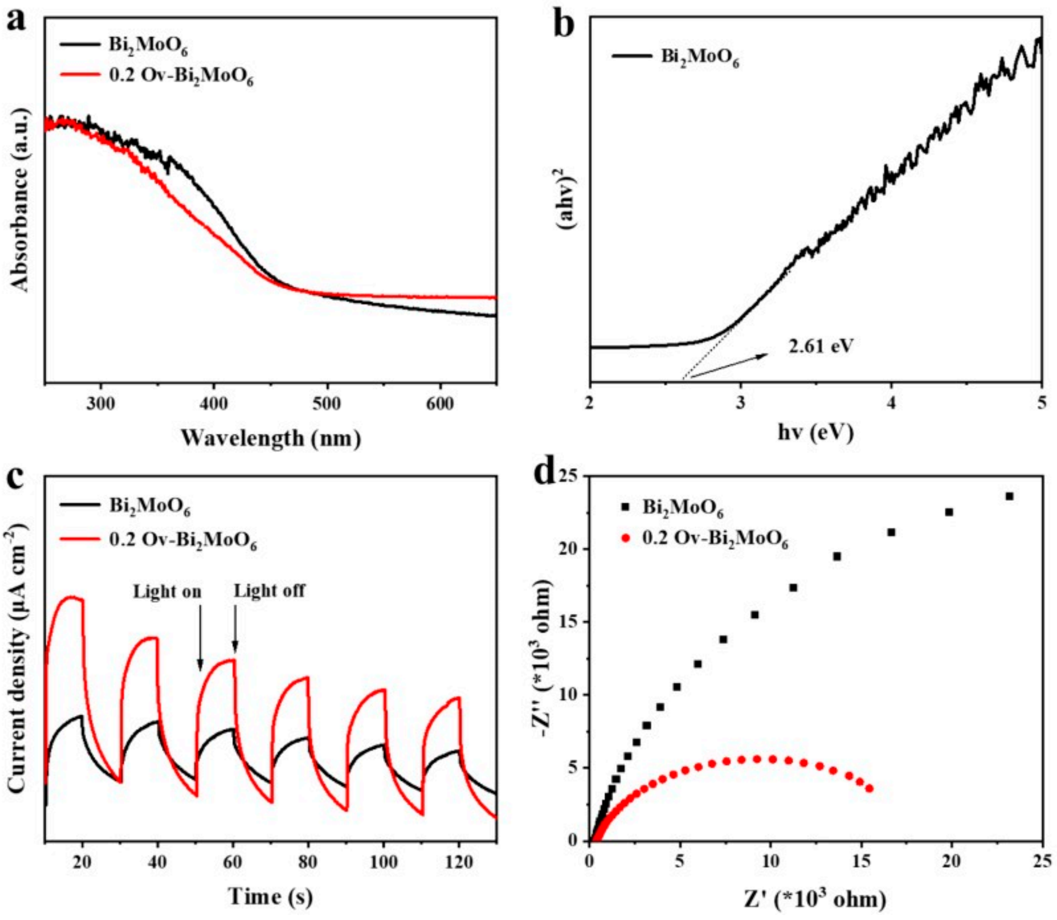

Figure S6. Bi2MoO6 and 0.2 Ov-Bi2MoO6: (a) UV–visible diffuse reflectance spectra, (b) bandgap plots, (c) transient photocurrent density changes with irradiation time, and (d) Nyquist impedance plots.

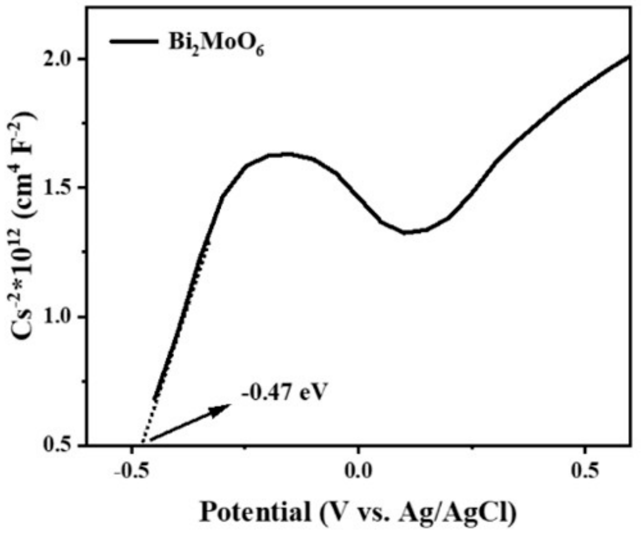

Figure S7. Mott-Schottky diagram of Bi_2MoO_6
